# Comparative finite element analysis of a novel distal tibiofibular locking plate versus conventional fixation methods for Tillaux-Chaput fractures

**DOI:** 10.3389/fbioe.2026.1904034

**Published:** 2026-09-03

**Authors:** Xiunian Hu, Fake Liao, Wutang Que, Huahui Huang, Han Li, Hui Liu, Weibin Lan, Rijiang Chen

**Affiliations:** 1 Department of Orthopaedics,Longyan First Affiliated Hospital of Fujian Medical University, Longyan, Fujian, China; 2 The Graduate School of Fujian Medical University, Fuzhou, Fujian, China; 3 Department of Clinical Laboratory Medicine, Longyan First Affiliated Hospital of Fujian Medical University, Longyan, Fujian, China

**Keywords:** biomechanics, distal tibiofibular syndesmosis, finite element analysis, internal fixation, locking plate, tillaux-chaput fracture

## Abstract

**Objective:**

Tillaux-Chaput fractures are avulsion fractures involving the tibial insertion of the anterior inferior tibiofibular ligament. Inadequate fixation may result in distal tibiofibular syndesmotic instability and post-traumatic ankle arthritis. Conventional fixation methods, such as cannulated screws and suture anchor fixation, exhibit inherent limitations in the management of small or comminuted fragments. This study aimed to compare the biomechanical performance of cannulated screw fixation, suture anchor fixation, and a newly developed distal tibiofibular locking plate system for Tillaux-Chaput fractures using finite element analysis.

**Methods:**

Computed tomography data from a healthy adult male ankle were used to reconstruct a three-dimensional finite element model consisting of the tibia, fibula, and talus. A Tillaux-Chaput fracture was simulated at the anterolateral distal tibia. Four fixation models were established: cannulated screw fixation, suture anchor fixation, and two configurations of the novel locking plate system. Five physiological loading conditions were simulated, including plantar flexion, dorsiflexion, internal rotation, external rotation, and single-leg standing. The maximum von Mises stress and total displacement of both the fracture fragment and fixation devices were evaluated.

**Results:**

Across all loading conditions, the novel locking plate configurations exhibited lower peak stress and displacement of the fracture fragment than both cannulated screw and suture anchor fixation. The plate-2 fixation demonstrated the most favorable overall performance, with fracture fragment stress ranging from 2.28 to 4.70 MPa under non-standing conditions and 4.63 MPa during single-leg standing. Under internal and external rotation, the cannulated screw model showed the highest fragment stress, reaching 30.72 and 29.42 MPa, respectively. During single-leg standing, the suture anchor fixation showed the highest fragment stress of 43.19 MPa. The locking plate models also exhibited a more uniform stress distribution and better control of fragment displacement, particularly under torsional loading.

**Conclusion:**

The novel distal tibiofibular locking plate system provided superior biomechanical stability compared with cannulated screw and suture anchor fixation in this finite element model of Tillaux-Chaput fracture. By combining plate support, locking screw fixation, and suture-assisted reinforcement, this system may offer a promising fixation strategy for small and comminuted Tillaux-Chaput fragments.

## 1 Introduction

Ankle injuries are common in clinical practice. Injuries to the lower tibiofibular junction accompanied by fractures account for approximately 1%–11% of all ankle injuries (Bragg et al., 2023; Rammelt and Obruba, 2015). A Tillaux-Chaput fracture is an avulsion fracture of the tibial insertion of the anterior tibiofibular ligament (Chaput’s tubercle). Inadequate treatment may lead to anterior tibiofibular ligament laxity and ankle joint space widening, carrying risks of chronic ankle instability and chronic post-traumatic arthritis (van Vlijmen et al., 2015; Yu et al., 2023). Therefore, anatomical reduction and stable fixation of the fracture fragment to restore normal tension in the anterior tibiofibular ligament and stability of the tibiofibular joint are essential for a favourable prognosis (Feng et al., 2018).

Currently, the commonly used methods of internal fixation for Tillaux-Chaput fractures include cannulated screws and suture anchor fixation. Cannulated screws fixation are relatively simple; however, when dealing with small and comminuted bone fragments, risks exist such as insufficient fixation strength, secondary fragment fragmentation, and impaired intraoperative drilling (Schepers et al., 2011; Desouky et al., 2021). Suture anchor fixation can be used to fix such small bone fragments, but its overall fixation strength is inferior to that of screws, and the cutting effect of the sutures on the bone fragments may lead to fixation failure and ligament laxity (Zhang et al., 2017). Fixation based on the tension band principle is relatively complex and places high demands on the status of bone fragments (Thaunat et al., 2007). Consequently, developing an internal fixation method capable of securely fixing small fracture fragments and effectively restoring stability of the tibiofibular joint remains an urgent clinical challenge.

The triangular support theory has been proven to provide excellent mechanical stability in the field of orthopaedic internal fixation (Ding et al., 2022; Yang et al., 2024). Based on this theory, we have developed a novel locking plate system for combined tibiofibular avulsion fractures. This plate system integrates multiple functions, including screw fixation and suture closure, aiming to provide a one-stop solution for Tillaux-Chaput fractures of varying sizes and degrees of comminution. Its core design concept is to integrate the mechanical effects of tension screws and sutures via the plate to form a stable composite fixation system.

This anatomically contoured low-profile plate is equipped with universal locking screw holes and reserved lateral suture channels, which enables combined fixation of locking screws and titanium sutures around the avulsed fragment rather than relying on single-point fixation only. Distinct from the cannulated screws and suture anchor fixation, the proposed plate distributes biomechanical loads across the tibial cortex via the plate framework, reduces peak stress within fracture fragments, and enhances resistance to shear and torsional force through suture reinforcement designs.

Finite Element Analysis (FEA), as a mature and reliable biomechanical research method, is widely applied in the performance evaluation and comparative studies of orthopaedic internal fixation devices (Wang et al., 2020; Li et al., 2019; Zhang et al., 2024). The advantages of this method include non-invasiveness, good reproducibility, and the ability to accurately calculate stress and strain at any node within the model. The aim of this study was to use FEA to systematically compare the biomechanical differences between cannulated screws, suture anchor fixation and our newly designed locked plate system for the tibiofibular joint in the fixation of Tillaux-Chaput fractures.

## 2 Materials and methods

### 2.1 Study design and procedure

This biomechanical comparative study was performed using finite element analysis. The entire workflow included CT data acquisition, three-dimensional skeletal reconstruction, fracture modelling, modelling and assembly of internal fixation devices, assignment of material properties, meshing, setting of boundary conditions, loading and simulation, and evaluation of biomechanical parameters. This study protocol has been approved by the First Longyan Hospital Affiliated to Fujian Medical University (Ethics Approval No.: LYREC2024-K040-01).

### 2.2 3D model construction and fracture modelling

One healthy adult male volunteer, aged 28 with a body mass index of 22.1 kg/m^2^, was recruited. The volunteer had no ankle injuries, deformities, tumours and other pathological conditions and signed an informed consent form. A 64-slice spiral CT scanner (Philips) was used to perform thin-slice scanning of the ankle joint (tube voltage, 120 kV; tube current, 150 mA; slice thickness, 0.625 mm), with the scanning range extending from 10 cm above the ankle joint plane to the sole of the foot. The raw DICOM data was imported into Mimics 21.0 software (Materialise, Belgium). Through operations such as grey-scale thresholding, region growing and hole filling, a preliminary three-dimensional geometric model comprising the tibia, fibula and talus was constructed and exported in STL file format.

The STL file was imported into Geomagic 2021 software (Geomagic, USA) to optimise the model’s surface. The model surface was smoothed using commands such as ‘Smooth’, ‘Denoise’ and ‘Remove Spikes’, whilst retaining key anatomical features of the skeleton. After verifying the model using the “Mesh Doctor”, the “Precision Surfaces” module was utilised to sequentially complete the steps of exploring contour lines, constructing surface patches, repairing surface patches, building a mesh, and fitting the surface. These procedures finally generated a high-quality solid model, which was saved as a STEP format file.

**FIGURE 1:**
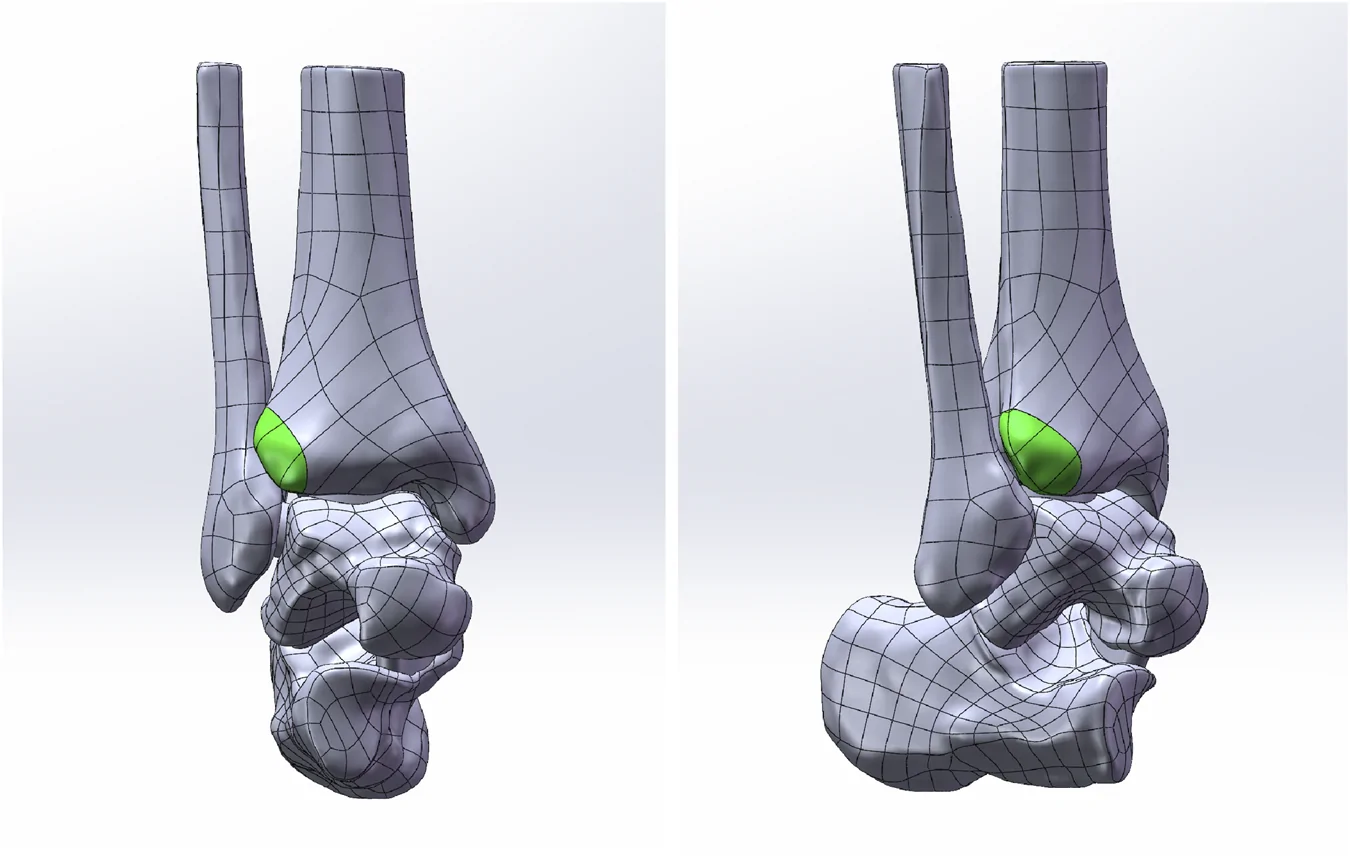
Three-dimensional reconstruction of the distal tibiofibular joint and simulated Tillaux-Chaput fracture model.

The STEP file was imported into SolidWorks 2021 software (Dassault Systèmes, France). First, the tibia and fibula were assembled at the origin in assembly mode. Referring to the characteristics of Tillaux-Chaput fractures described in clinical reports and the literature (Yu et al., 2023; Feng et al., 2018), the ‘Split’ command was used to create a fracture line on the anterolateral aspect of the distal tibia. This procedure simulated the formation of a typical avulsion fragment to establish the fracture model (Figure 1).

**FIGURE 2:**
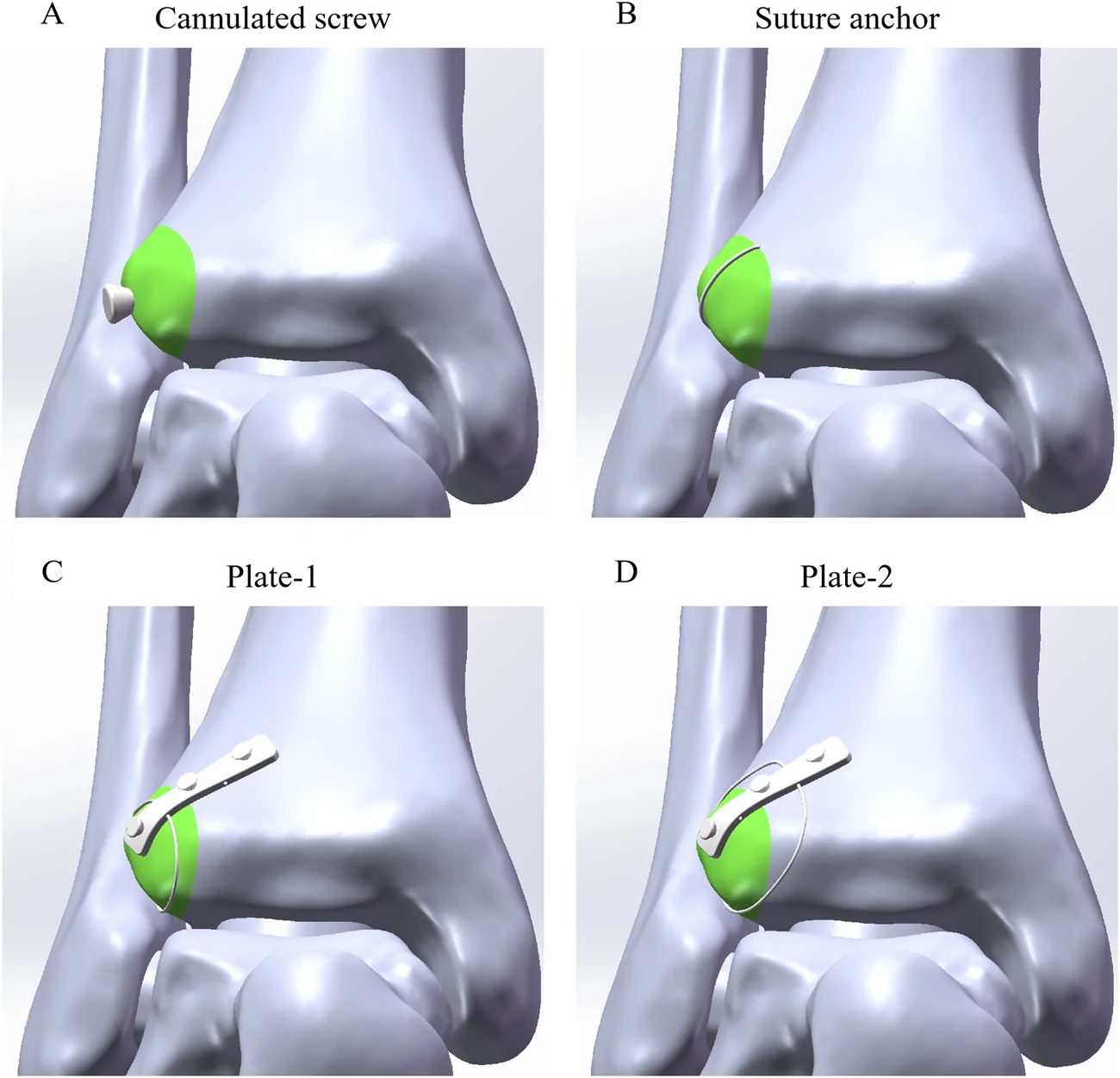
Three-dimensional models of the four fixation constructs used for Tillaux-Chaput fracture fixation. Four fixation models were established and assembled with the same Tillaux-Chaput fracture model. **(A)** Cannulated screw fixation using a single cannulated compression screw. **(B)** Suture anchor fixation using a metal rivet and tail wire. **(C)** Plate-1 fixation, proximal-suture fixation, consisting of an anatomically contoured distal tibiofibular locking plate, locking screws, and suture reinforcement through the first lateral hole. **(D)** Plate-2 fixation, distal-suture fixation, in which an additional suture pathway through the second lateral hole was used to reinforce fixation and improve load redistribution around the fracture fragment.

Subsequently, based on clinical specimens and surgical requirements, three sets of three-dimensional models of internal fixation devices were constructed (Figure 2).

1. Cannulated screw fixation: Simulating a single hollow compression screw inserted perpendicular to the fracture line, with a screw diameter of 3.0 mm, a head diameter of 5.0 mm, and a hollow section diameter of 2.5 mm.
2. Suture anchor fixation: Simulates fixation using a single wire-tipped anchor rivet; the rivet has a diameter of 2.3 mm and the tail wire a diameter of 0.5 mm. The suture anchor model used a metallic rivet with a trailing suture with a trailing suture used to capture and compress the avulsed fragment.
3. Locking plate model: This novel technology is based on a patented design. The plate features an anatomical low-notch design, with a thickness of 1.0 mm, a width of 4.25 mm, and a length adapted to the fracture site. The plate is designed with 1.5 mm diameter universal locking screw holes, channels for passing micro-lateral hook titanium cables, and suture holes. The distal tibiofibular locking plate is anatomically contoured along the anterolateral distal tibia to follow the course of the Tillaux-Chaput fragment. Sutures are passed around the avulsion fragment and secured through the preset plate holes. This allows the fragment to be indirectly compressed against the tibial surface via the plate, rather than relying solely on direct screw fixation. We ultimately assembled two plate-suture modes to demonstrate their functional versatility: Plate and proximal-suture fixation (Plate one fixation) involves sutures passing through the first pre-set lateral hole to hook the fracture fragments after the plate has been fixed; Plate and distal-suture fixation (Plate 2 fixation), which is similar to plate and proximal-suture fixation, with sutures passing through the second pre-set lateral hole to hook the fracture fragments, thereby achieving reinforced fixation of the anterior tibiofibular ligament. Both modes were analysed with the fracture model.

### 2.3 Material Properties, Contact Settings, Meshing and validation

The assembled model was imported into Ansys Workbench 2021 R1 (ANSYS Inc., USA). All materials are assumed to be homogeneous, isotropic and linearly elastic. Material parameters such as the elastic modulus and Poisson’s ratio for bone, cartilage and internal fixations were assigned based on previous literature (Huang et al., 2023; Liu et al., 2020); specific values are shown in Table 1. This linear elastic simplification eliminates confounding nonlinear material behaviors and guarantees consistent comparative conditions across all four fixation constructs in static biomechanical analysis.

**TABLE 1:** Material properties of bones, cartilage, implants and sutures in finite element models.

Materials	Modulus of elasticity (MPa)	Poisson’s ratio
Cortical bone	17,000	0.3
spongy bone	700	0.3
cartilage	10	0.4
cannulated screw (titanium alloy)	110,000	0.3
Titanium suture anchor	110,000	0.3
Anchor suture (0.5 mm diameter)	11,000	0.26
Titanium plate	110,000	0.3
Screw	110,000	0.3
suture (0.4 mm)	2250	0.3

The ligaments were modelled using linear spring elements, with spring stiffness coefficients reported by others (Blank et al., 2022; Alastuey-López et al., 2021); the specific values are shown in Table 2. Linear spring elements adequately reproduce ligament tensile restraint under static loading while significantly reducing computational time for multi-model comparative screening.

**TABLE 2:** Spring stiffness of ankle ligaments in finite element models.

Ligament	Spring stiffness (N/mm)
Anterior inferior tibiofibular ligament (AITFL)	90
Posterior inferior tibiofibular ligament (PITFL)	90
Anterior talofibular ligament (ATFL)	90
Posterior talofibular ligament (PTFL)	70
Calcaneofibular ligament (CFL)	70
Anterior tibiotalar ligament	70
Posterior tibiotalar ligament	80
Tibiocalcaneal ligament	122
Interosseous membrane (IOM)	400

Cartilage-to-cartilage contact was modeled as frictional contact with a coefficient of 0.01. The fracture interface was assigned Coulomb friction with a coefficient of 0.46 (Eberl et al., 2009). The locking interfaces between plates and screws, as well as screw-bone engagement regions, were defined as bonded contacts to simulate rigid fixation. Other implant-bone interfaces were modeled as frictional contacts with a coefficient of 0.30 (Li et al., 2019). Bonded constraints were applied only for locking screw-plate and screw-bone threaded interfaces to mimic rigid mechanical interlock, while frictional contact was adopted for all free sliding interfaces to replicate natural relative micro-movement between tissues and implants.

Tetrahedral solid elements (SOLID187) were used to mesh all model components. To ensure computational accuracy and convergence, the mesh was refined in the fracture region and around the internal fixators. Mesh sensitivity analysis indicated that when the mesh size was refined to the current scheme, the variation in results was less than 5%. The final numbers of nodes and elements for each model are summarised in Table 3.

**TABLE 3:** Nodes and elements of four fixation models after mesh convergence.

Model	Cannulated screw	Suture anchor fixation	Plate-1 fixation	Plate-2 fixation
Number of nodes	638,555	652,111	707,484	730,988
Number of elements	428,558	415,187	468,147	482,684

In this study, the validation of the ankle joint model employed the experimental method described by Anderson et al.: a load of 600 N was applied to the tibia and fibula, while the distal end of the calcaneus was fully immobilized, in order to observe the contact pressure distribution at the tibiofibular joint (Anderson et al., 2007).

### 2.4 Boundary conditions and loading conditions

To simulate the forces on the ankle joint during physiological movement, five loading conditions were established according to the previous studies (Talbott et al., 2023; Takahashi et al., 2025). For plantar flexion, dorsiflexion, internal rotation and external rotation loading conditions, the proximal cross-section of the tibia and fibula was fixed, and a torque of 1.7 N·m was applied to the talar surface in the directions of plantar flexion, dorsiflexion, internal rotation and external rotation, respectively; for the single-leg stance condition, the calcaneal base was fixed, and concentrated vertical downward forces of 500 N and 100 N were imposed on the proximal cross-sections of the tibia and fibula to simulate the physiological axial load distribution with a tibia-to-fibula load ratio of 5:1.

### 2.5 Primary outcome measures and data analysis

The key outcome metrics of this analysis include.

1. Maximum von Mises stress (MPa) on the fracture fragments, used to assess the load-bearing capacity of the fragments and the risk of potential failure.
2. Maximum total displacement (mm) of the fracture fragments, used to assess the stability of the fixation.
3. Maximum von Mises stress (MPa) on the internal fixation devices, used to assess the risk of fatigue and fracture in the devices.
4. Maximum total displacement (mm) of the internal fixation devices. Due to the univariate nature of finite element analysis outputs, this study directly compares the quantitative results of the models across groups, without the need for hypothesis testing based on statistical inference from samples.

## 3 Results

**FIGURE 3:**
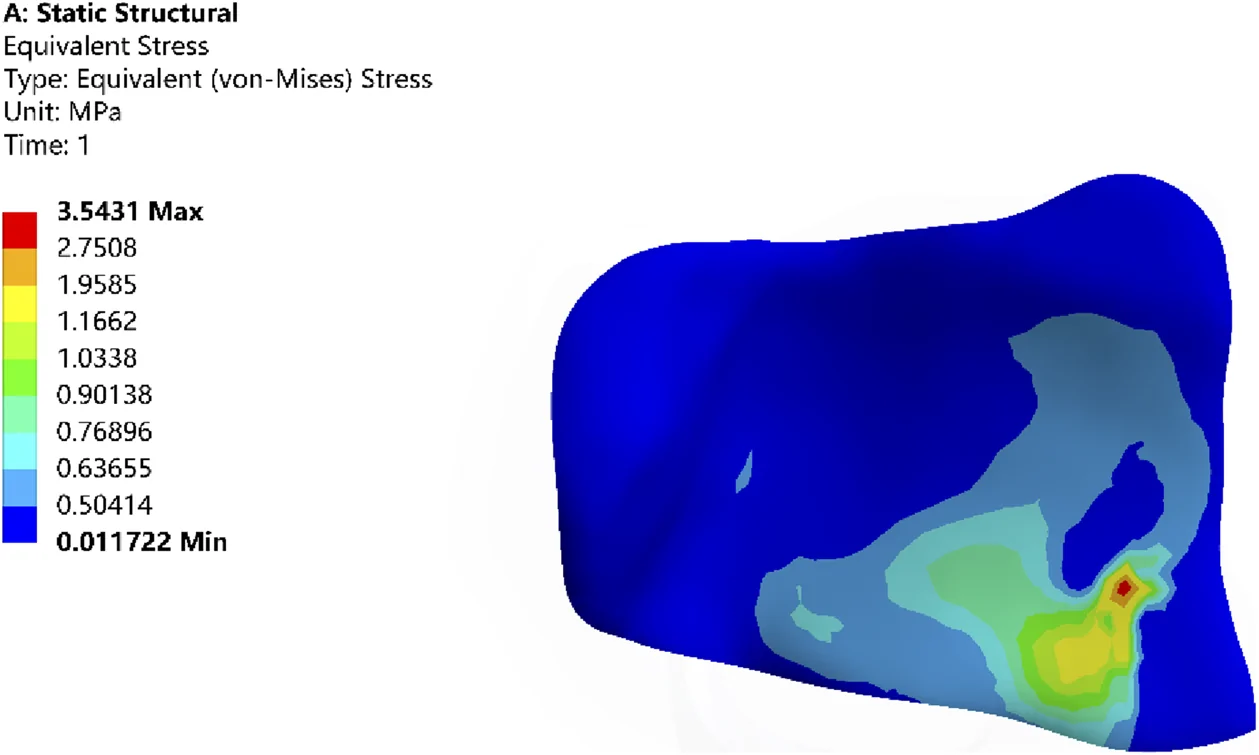
Contour distribution of equivalent von Mises contact stress on the distal tibial articular surface under 600 N vertical axial compressive load for model validation. The peak contact stress of 3.5431 MPa concentrates at the anterolateral region of the joint surface, consistent with published cadaver biomechanical findings.

Prior to comparing biomechanical indicators across four fixation constructs, we conducted model validation under 600 N axial vertical load. The simulated peak tibiotalar contact pressure of 3.5431 MPa closely matched the cadaver experimental reference value of 3.690 MPa, with relative deviation less than 5% (Figure 3).

### 3.1 Von Mises stress in fracture fragments

The peak von Mises stress in the fracture fragment varied across the four fixation constructs and loading conditions. Overall, the two locking plate configurations exhibited lower peak stresses in the fracture fragment than the cannulated screw and suture anchor fixation constructs under all simulated loading conditions (Table 4).

**TABLE 4:** Peak von Mises stress of fracture fragment under five loading conditions (MPa).

Loading conditions	Cannulated screw	Suture anchor fixation	Plate-1 fixation	Plate-2 fixation
Plantar flexion	7.8323	7.1606	3.6955	3.3459
Dorsiflexion	7.1203	6.1611	2.7252	2.2769
Internal rotation	30.723	13.697	5.1509	4.6051
External rotation	29.416	20.933	4.9615	4.6981
Standing position	20.838	43.189	6.9827	4.6281

Under plantar flexion, the peak stress in the fracture fragment was 7.832 MPa in the cannulated screw and 7.161 MPa in the suture anchor fixation. In comparison, the values decreased to 3.696 MPa in the plate-1 fixation and 3.346 MPa in the plate-2 fixation. A similar trend was observed under dorsiflexion, with peak stresses of 7.120 MPa, 6.161 MPa, 2.725 MPa, and 2.277 MPa in the cannulated screw fixation, suture anchor fixation, plate-1 fixation, and plate-2 fixation, respectively.

**FIGURE 4:**
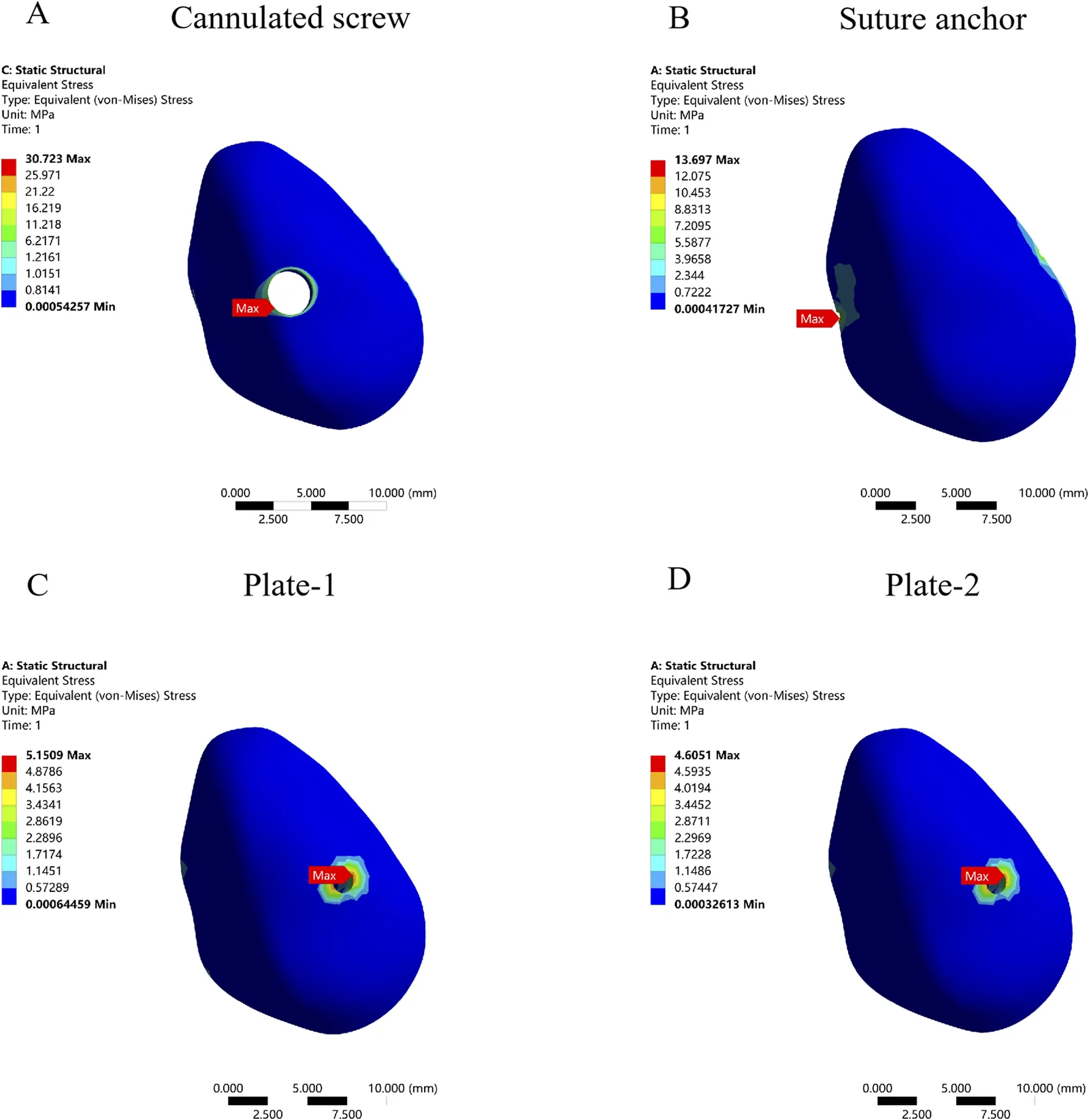
Von Mises stress distribution in the fracture fragment under internal rotation. The contour plots show the stress distribution in the Tillaux-Chaput fracture fragment under internal rotation loading. **(A)** Cannulated screw group. **(B)** Suture anchor fixation group. **(C)** Plate-1 fixation. **(D)** Plate-2 fixation. The cannulated screw construct showed evident stress concentration around the screw-bone interface near the fracture line. In contrast, both locking plate configurations showed a more distributed stress pattern, with plate-2 producing the lowest peak stress in the fracture fragment.

The difference between the fixation constructs became more evident under rotational loading. During internal rotation, the cannulated screw showed the highest peak stress in the fracture fragment, reaching 30.723 MPa. The corresponding values were 13.697 MPa in the suture anchor fixation, 5.151 MPa in the plate-1 fixation, and 4.605 MPa in the plate-2 fixation (Figure 4). During external rotation, the peak stresses were 29.416 MPa, 20.933 MPa, 4.962 MPa, and 4.698 MPa, respectively. These findings indicate that the locking plate constructs, particularly plate-2, reduced stress concentration in the fracture fragment under torsional loading.

**FIGURE 5:**
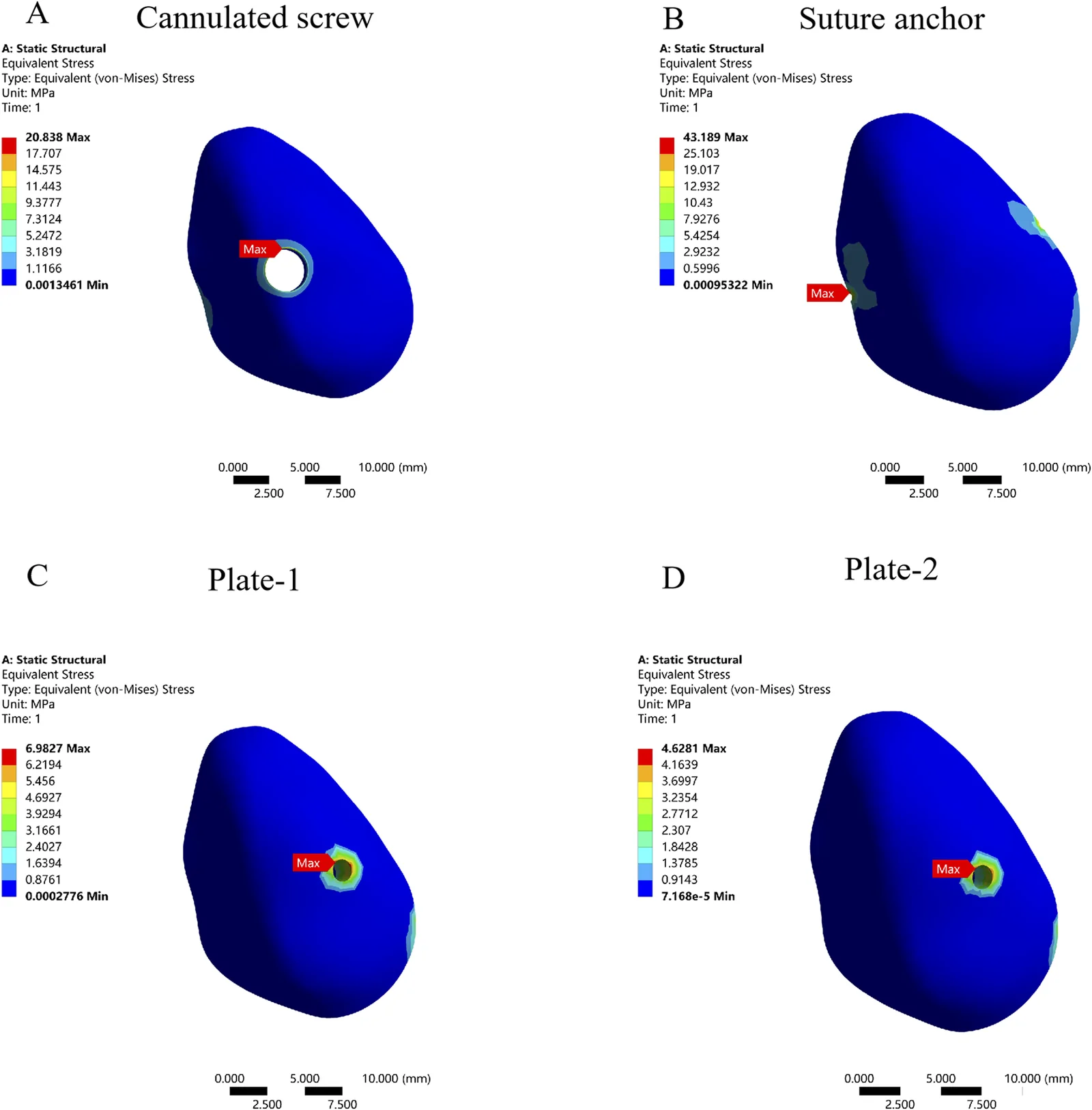
Von Mises stress distribution in the fracture fragment under single-leg standing. The contour plots show the stress distribution in the fracture fragment under simulated single-leg standing. **(A)** Cannulated screw group. **(B)** Suture anchor fixation group. **(C)** Plate-1 fixation. **(D)** Plate-2 fixation. A marked local stress concentration was observed around the rivet insertion region in the suture anchor fixation group. The locking plate groups showed lower and more widely distributed stresses within the fracture fragment, particularly in plate-2.

Under the single-leg standing condition, the suture anchor fixation group showed the highest fracture fragment stress, with a peak value of 43.189 MPa, followed by the cannulated screw at 20.838 MPa. In contrast, the peak stresses in the plate-1 fixation and the plate-2 fixation were 6.983 MPa and 4.628 MPa, respectively (Figure 5). The stress contour plots showed marked local stress concentration around the rivet insertion site in the suture anchor fixation group, whereas the locking plate groups demonstrated a more distributed stress pattern across the fixation region.

### 3.2 Displacement of fracture fragments

The maximum displacement of the fracture fragment was generally small under plantar flexion, dorsiflexion, internal rotation, and external rotation. However, differences among the fixation constructs were observed (Table 5).

**TABLE 5:** Maximum fragment total displacement under five loading conditions (mm).

Operating conditions	Cannulated screw	Suture anchor fixation	Plate-1 fixation	Plate-2 fixation
Plantar flexion	0.033666	0.029561	0.031760	0.030641
Dorsiflexion	0.053965	0.036701	0.031782	0.030816
Internal rotation	0.025263	0.024232	0.023121	0.020095
External rotation	0.032633	0.024108	0.024341	0.021546
Standing position	0.74483	0.65576	0.62589	0.63078

Under plantar flexion, the maximum displacement values were 0.0337 mm in the cannulated screw, 0.0296 mm in the suture anchor fixation group, 0.0318 mm in the plate-1 fixation, and 0.0306 mm in the plate-2 fixation. Under dorsiflexion, the cannulated screw showed the greatest displacement, at 0.0540 mm, whereas the values in the suture anchor fixation group, plate-1 fixation, and plate-2 fixation were 0.0367 mm, 0.0318 mm, and 0.0308 mm, respectively.

**FIGURE 6:**
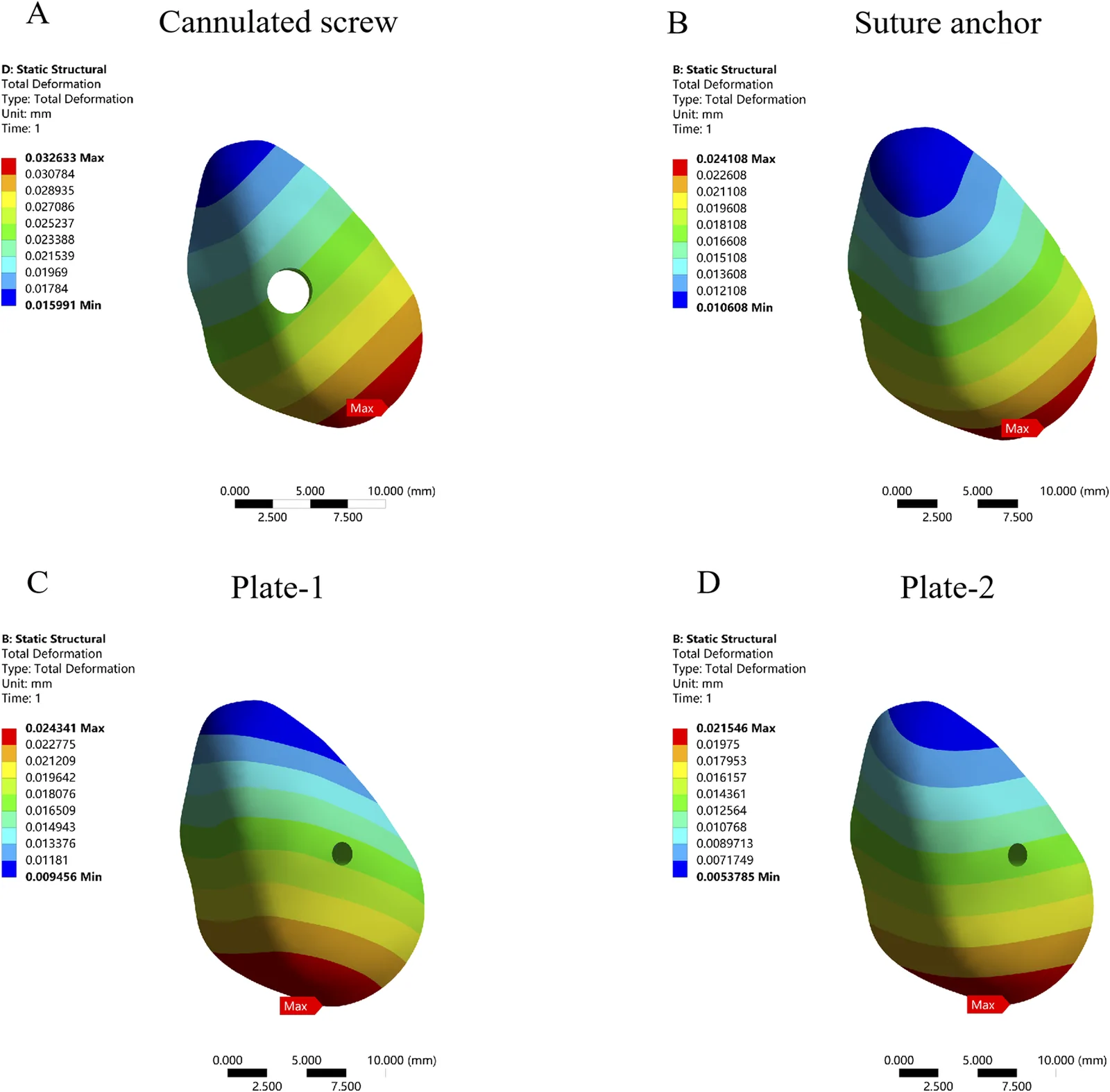
Total displacement distribution of the fracture fragment under external rotation. The contour plots show the displacement distribution of the Tillaux-Chaput fracture fragment under external rotation loading. **(A)** Cannulated screw group. **(B)** Suture anchor fixation group. **(C)** Plate-1 fixation. **(D)** Plate-2 fixation. The cannulated screw showed greater displacement at the distal margin of the fracture fragment. The locking plate constructs demonstrated smaller and more uniform displacement, with plate-2 fixation showing the lowest maximum displacement under this loading condition.

Under internal rotation, the displacement values were 0.0253 mm, 0.0242 mm, 0.0231 mm, and 0.0201 mm in the cannulated screw, suture anchor fixation, plate-1 fixation, and plate-2 fixation groups, respectively. Under external rotation, the cannulated screw again showed the greatest displacement, at 0.0326 mm, while plate-2 fixation showed the lowest value, at 0.0215 mm (Figure 6).

**FIGURE 7:**
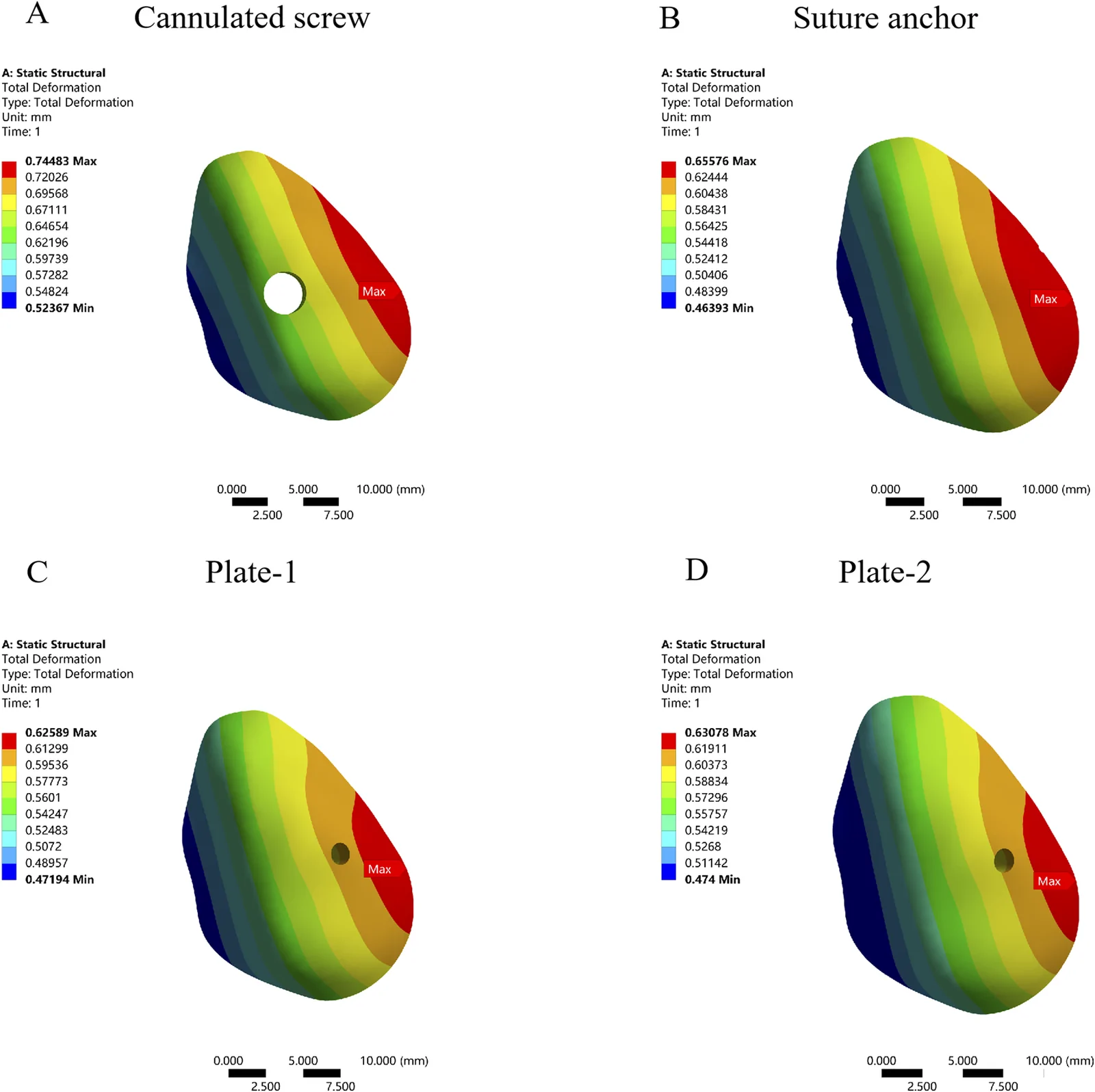
Total displacement distribution of the fracture fragment under single-leg standing. The contour plots show the displacement of the fracture fragment under simulated full weight-bearing on one leg. **(A)** Cannulated screw group. **(B)** Suture anchor fixation group. **(C)** Plate-1 fixation. **(D)** Plate-2 fixation. All fixation constructs showed increased displacement under axial loading. The cannulated screw showed the largest fragment displacement, whereas both locking plate configurations showed lower displacement and a more continuous displacement distribution across the fracture region.

During single-leg standing, the displacement of the fracture fragment increased markedly in all groups compared with non-weight-bearing loading conditions. The cannulated screw showed the largest displacement, at 0.7448 mm, followed by the suture anchor fixation group at 0.6558 mm. The values in plate-1 fixation and plate-2 fixation were 0.6259 mm and 0.6308 mm, respectively (Figure 7). Although the difference between the two plate configurations was small under this condition, both plate constructs showed lower fracture fragment displacement than the cannulated screw construct.

### 3.3 Von Mises stress in the internal fixation devices

The stress distribution within the fixation devices differed substantially among the four constructs (Table 6).

**TABLE 6:** Peak von Mises stress of implants under five loading conditions (MPa).

Operating Conditions	Cannulated screw	Suture anchor fixation	Plate-1 fixation	Plate-2 fixation
Plantar flexion	11.322	6.6632	7.0296	6.7927
Dorsiflexion	15.17	6.675	6.9498	6.7508
Internal rotation	46.843	18.543	20.227	20.224
External rotation	42.793	37.467	34.973	26.428
Standing position	19.652	45.769	48.171	34.678

Under plantar flexion, the cannulated screw showed the highest implant stress, with a peak value of 11.322 MPa. The corresponding stresses in the suture anchor fixation group, plate-1 fixation, and plate-2 fixation were 6.663 MPa, 7.030 MPa, and 6.793 MPa, respectively. Under dorsiflexion, the cannulated screw again showed the highest value, at 15.170 MPa, whereas the other three groups showed similar lower values ranging from 6.675 to 6.950 MPa.

**FIGURE 8:**
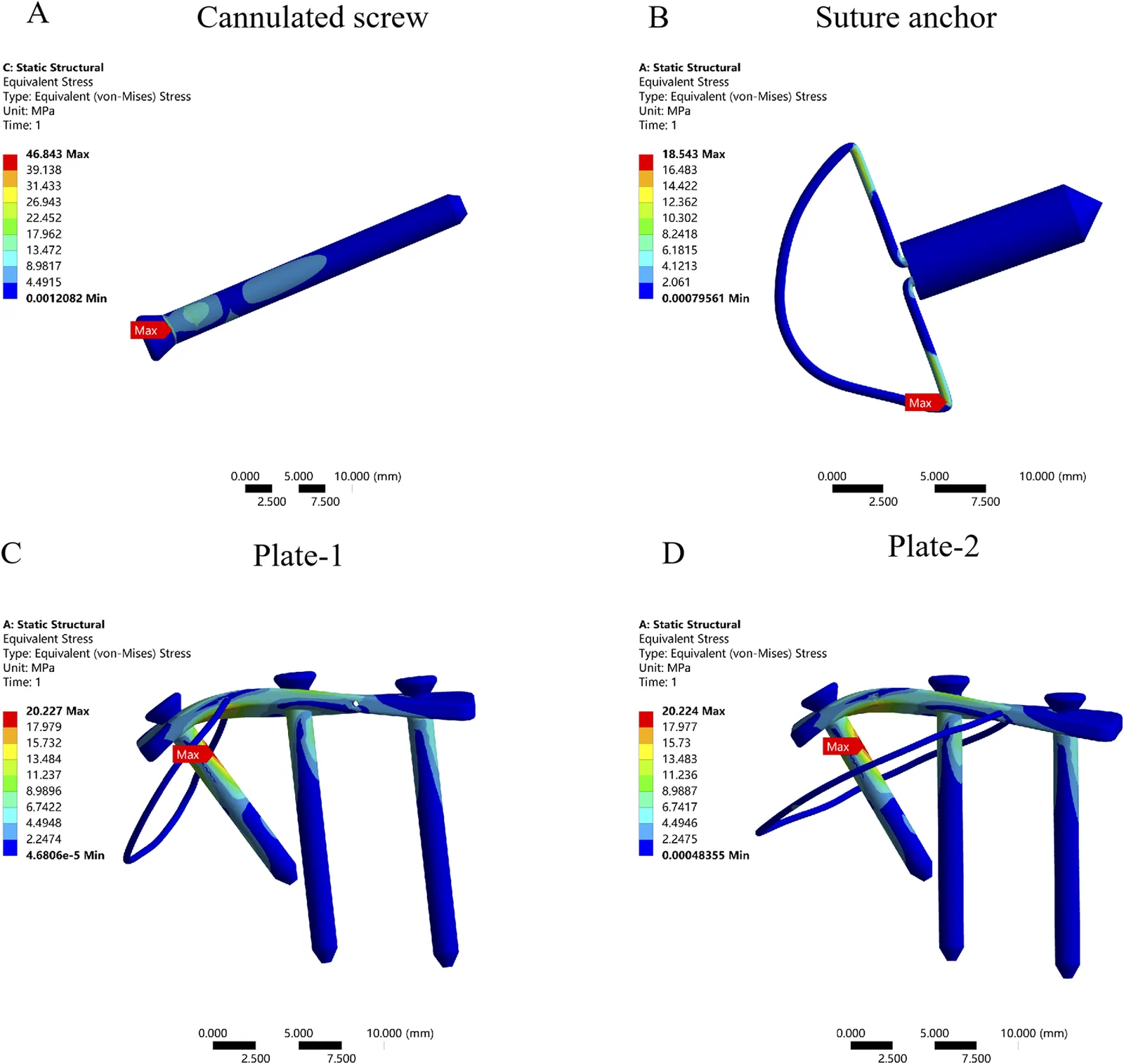
Von Mises stress distribution in the internal fixation devices under internal rotation. The contour plots show the stress distribution within the fixation devices under internal rotation loading. **(A)** Cannulated screw group. **(B)** Suture anchor fixation group. **(C)** Plate-1 fixation. **(D)** Plate-2 fixation. The cannulated screw construct showed stress concentration along the screw shaft near the fracture line. In the suture anchor fixation group, stress was mainly concentrated near the rivet-wire junction. In the locking plate groups, stress was distributed along the plate-screw structure, with configuration 2 showing a more even stress pattern.

Rotational loading produced higher implant stresses, particularly in the cannulated screw. During internal rotation, the peak implant stress reached 46.843 MPa in the cannulated screw. The values in the suture anchor fixation group, plate-1 fixation, and plate-2 fixation were 18.543 MPa, 20.227 MPa, and 20.224 MPa, respectively (Figure 8). During external rotation, the peak implant stress was 42.793 MPa in the cannulated screw, 37.467 MPa in the suture anchor fixation group, 34.973 MPa in plate-1 fixation, and 26.428 MPa in plate-2 fixation.

**FIGURE 9:**
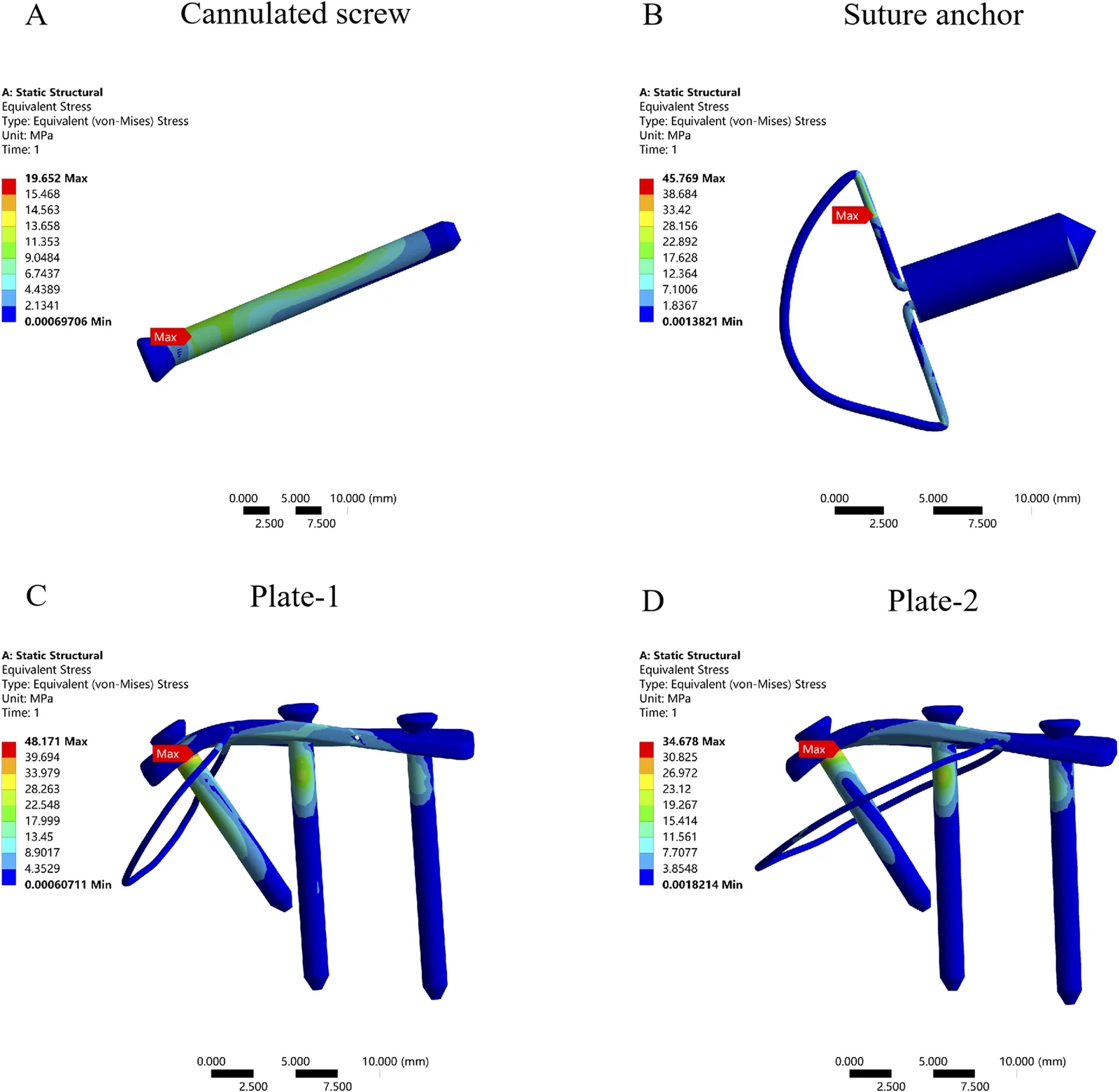
Von Mises stress distribution in the internal fixation devices under single-leg standing. The contour plots show the implant stress distribution under simulated single-leg standing. **(A)** Cannulated screw group. **(B)** Suture anchor fixation group. **(C)** Plate-1 fixation. **(D)** Plate-2 fixation. Plate-1 fixation showed stress concentration around the plate-screw interface. Compared with plate-1 fixation, plate-2 fixation showed lower peak stress, suggesting that the additional suture pathway contributed to load redistribution within the fixation construct.

Under the single-leg standing condition, plate-1 fixation showed the highest implant stress, at 48.171 MPa. The stress was mainly concentrated around the plate-screw interface. In contrast, plate-2 fixation showed a lower peak implant stress of 34.678 MPa, suggesting that the additional suture pathway contributed to load redistribution within the construct. The suture anchor fixation group showed a peak stress of 45.769 MPa, whereas the cannulated screw showed a lower implant stress of 19.652 MPa. However, this lower implant stress in the cannulated screw should be interpreted together with the relatively large fracture fragment displacement observed under the same loading condition (Figure 9).

### 3.4 Displacement of the internal fixation devices

The maximum displacement of the fixation devices showed a pattern similar to that of the fracture fragment displacement (Table 7).

**TABLE 7:** Maximum implant total displacement under five loading conditions (mm).

Operating conditions	Cannulated screw	Suture anchor fixation	Plate-1 fixation	Plate-2 fixation
Plantar flexion	0.031683	0.026469	0.031578	0.030856
Dorsiflexion	0.052011	0.033056	0.031790	0.031012
Internal rotation	0.033425	0.024034	0.025807	0.024755
External rotation	0.032687	0.023873	0.025566	0.024593
Standing position	0.70213	0.65256	0.68415	0.67544

Under plantar flexion, the implant displacement values were close among the four groups, ranging from 0.0265 mm to 0.0317 mm. Under dorsiflexion, the Cannulated screw showed the greatest displacement, at 0.0520 mm, while the values in the suture anchor fixation group, plate-1 fixation, and plate-2 fixation were 0.0331 mm, 0.0318 mm, and 0.0310 mm, respectively.

Under internal rotation, the implant displacement values were 0.0334 mm in the cannulated screw, 0.0240 mm in the suture anchor fixation group, 0.0258 mm in plate-1 fixation, and 0.0248 mm in plate-2 fixation. Under external rotation, the corresponding values were 0.0327 mm, 0.0239 mm, 0.0256 mm, and 0.0246 mm, respectively.

**FIGURE 10:**
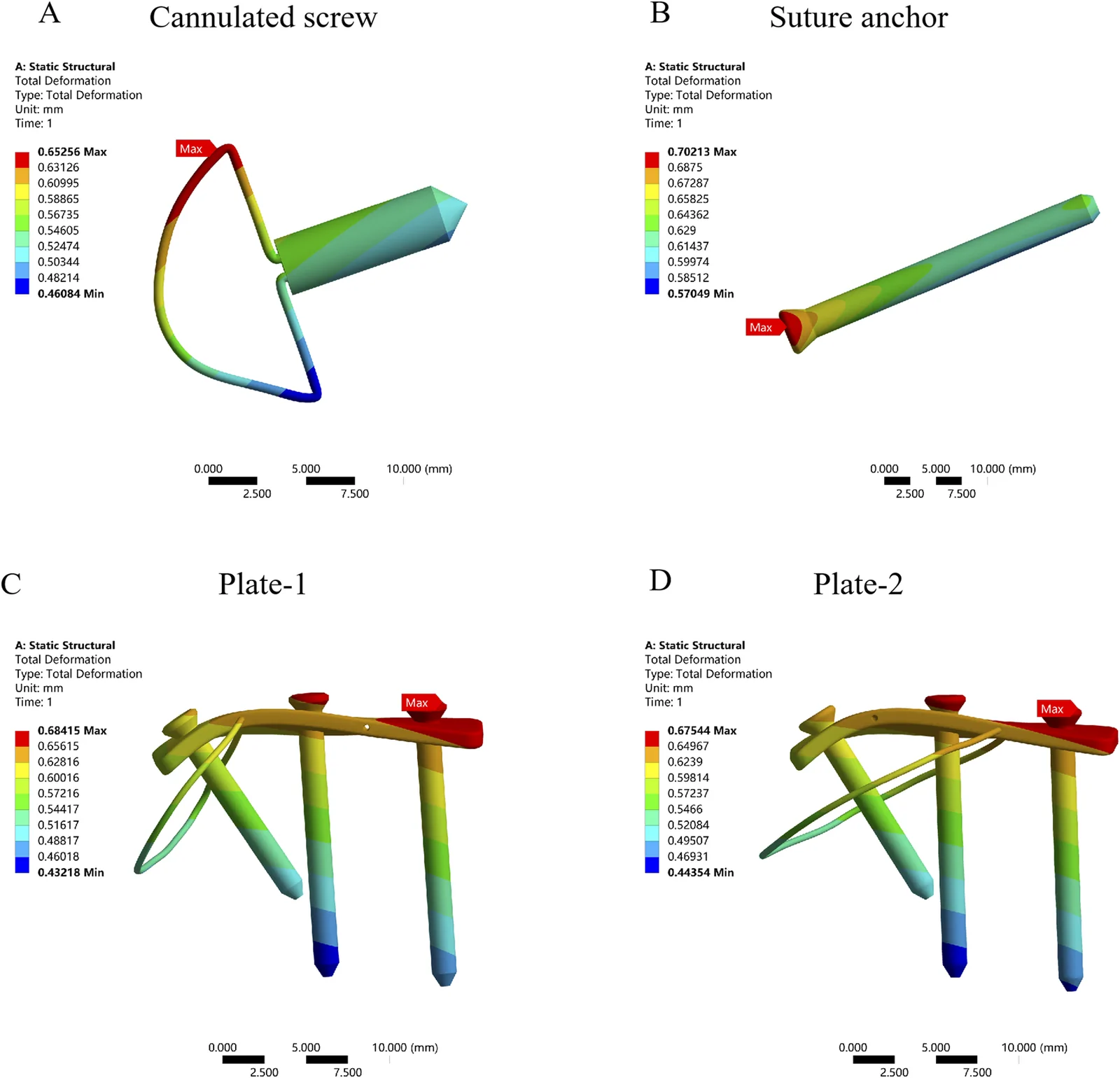
Total displacement distribution of the internal fixation devices under single-leg standing. The contour plots show the displacement distribution of the fixation devices under simulated single-leg standing. **(A)** Cannulated screw group. **(B)** Suture anchor fixation group. **(C)** Plate-1 fixation. **(D)** Plate-2 fixation. The cannulated screw showed a displacement gradient along the screw axis, suggesting bending deformation of the screw construct. The locking plate groups showed a more continuous displacement distribution along the plate and screw components, with plate-2 showing slightly lower implant displacement than plate-1.

During single-leg standing, all constructs showed increased implant displacement. The cannulated screw showed the largest displacement, at 0.7021 mm, whereas the suture anchor fixation group showed the lowest value, at 0.6526 mm. The displacement values of plate-1 fixation and plate-2 fixation were 0.6842 mm and 0.6754 mm, respectively (Figure 10). The displacement contour plots showed that the cannulated screw construct tended to deform along the screw axis, while the plate constructs showed a more continuous displacement distribution along the plate and screw components.

## 4 Discussion

Tillaux-Chaput fractures involve the anterolateral margin of the distal tibia and are closely related to the tibial attachment of the anterior inferior tibiofibular ligament. Although these fractures are often small in size, inadequate reduction or unstable fixation may affect distal tibiofibular syndesmosis stability and ankle joint congruity. For small or comminuted fragments, conventional screw fixation can be technically challenging because the screw trajectory and purchase are limited by the size and morphology of the fragment (Bragg et al., 2023; Yu et al., 2023; Ogilvie-Harris et al., 1994a). In the present finite element study, we compared cannulated screw fixation, suture anchor fixation, and two configurations of a distal tibiofibular locking plate system for Tillaux-Chaput fracture fixation. The main finding was that the locking plate constructs, particularly plate-2 fixation, reduced stress concentration in the fracture fragment and provided more stable displacement control under most simulated loading conditions. Importantly, the contact pressure distribution and peak magnitude of our ankle FE model under axial vertical loading are highly consistent with cadaveric biomechanical measurements reported by Anderson et al., which further confirms that our geometric reconstruction, material assignments and contact settings can reliably reproduce physiological ankle mechanical responses (Anderson et al., 2007).

The differences among the four constructs were most evident under rotational loading. During internal and external rotation, the cannulated screw model showed higher peak von Mises stress in both the fracture fragment and the implant (Rammelt and Obruba, 2015; Clanton et al., 2017). This finding is mechanically reasonable because single-screw fixation provides compression mainly along the screw axis, while its ability to resist rotational shear depends largely on the screw-bone interface and the quality of fragment purchase (Ogilvie-Harris et al., 1994b). When rotational loading is applied, the fracture fragment may tend to rotate around the screw axis, resulting in local stress concentration near the fracture line and around the screw passage. This pattern was also reflected in the displacement distribution, where the cannulated screw construct showed a relatively concentrated deformation pattern (Lee et al., 2021). These findings suggest that, although screw fixation is simple and widely used, its mechanical performance may be less favorable when the fracture fragment is small or when rotational stability is required (Nelson et al., 2010).

The suture anchor fixation construct showed a different mechanical response. Compared with the cannulated screw construct, suture anchor fixation reduced fracture fragment stress under internal rotation and showed relatively small displacement under several low-load conditions. However, under simulated single-leg standing, this construct produced the highest peak stress in the fracture fragment. The stress contour showed local stress concentration around the rivet insertion region, suggesting that the load was transferred through a relatively small contact area. This may increase the risk of local bone stress concentration, especially when the fragment is thin or osteoporotic. Previous studies on ankle ligament mechanics and syndesmotic stabilization have shown that load transfer across the ankle is strongly influenced by the direction of loading and by the local stiffness of ligamentous and fixation structures (Siegler et al., 1988). Therefore, suture anchor fixation may provide useful soft-tissue-based stabilization, but its mechanical behavior appears to be sensitive to the local bone-implant interface and the direction of loading.

In contrast, the two locking plate configurations showed lower fracture fragment stress across the five loading conditions. This result can be explained by the broader load-sharing structure created by the plate, locking screws, and suture pathway. Unlike single-point fixation, the plate construct distributes load through multiple fixation points and transfers part of the mechanical demand from the fracture fragment to the surrounding distal tibial cortex (Ding et al., 2022; Perren, 2022). This reduces direct stress concentration in the small anterolateral fragment and may be particularly useful for fragments that are too small to achieve stable screw purchase. In addition, the plate design allows fixation to be placed around the fragment rather than entirely through it, which may help preserve fragment integrity during fixation (Yang et al., 2024; Clanton et al., 2017).

Between the two plate configurations, plate-2 fixation showed the lowest fracture fragment stress in all loading conditions. The difference between the two configurations was particularly relevant under torsional loading and simulated single-leg standing. In plate-2 fixation, the additional suture pathway appeared to improve load redistribution around the fracture region. This may have reduced the stress transmitted directly to the fracture fragment and decreased peak stress at the implant-bone interface. Although the displacement difference between the two plate configurations was small under some conditions, plate-2 fixation provided a more balanced mechanical response, with low fracture fragment stress, acceptable implant stress, and relatively small displacement. From a biomechanical perspective, this suggests that the additional suture pathway may contribute to construct stability rather than simply increasing fixation complexity (Weng et al., 2020; Miller et al., 2010).

The implant stress results also deserve attention. Under single-leg standing, plate-1 fixation showed the highest implant stress, with stress mainly concentrated around the plate-screw interface. This may indicate that when the suture pathway is insufficient to share load, more stress is transferred to the plate-screw interface. By comparison, plate-2 fixation showed lower implant stress under the same loading condition, suggesting that the additional suture pathway helped share the axial load. However, implant stress should not be interpreted in isolation. For example, the cannulated screw showed relatively low implant stress under single-leg standing, but it also showed the largest fracture fragment displacement. This indicates that a lower implant stress value does not necessarily represent better fixation; rather, implant stress should be evaluated together with fracture fragment stress and displacement (Ding et al., 2022).

The displacement results provide further insight into construct stability. Under plantar flexion, dorsiflexion, internal rotation, and external rotation, all constructs showed relatively small fragment displacement. This suggests that under low-load or controlled loading conditions, each fixation method could maintain basic fragment stability in the model. However, during simulated single-leg standing, fragment displacement increased in all groups, reflecting the greater mechanical demand of axial weight-bearing. Under this condition, the cannulated screw construct showed the largest fragment displacement, whereas the plate constructs maintained lower displacement. This finding supports the view that multi-point fixation and load redistribution may be beneficial when early mechanical stability is required (Zhang et al., 2024; Stauffer et al., 1977). Nevertheless, these results should be interpreted as comparative mechanical trends rather than direct evidence for immediate postoperative weight-bearing.

The present findings have potential clinical implications. For Tillaux-Chaput fractures with a sufficiently large fragment, cannulated screw fixation remains a practical option because it is simple and minimally invasive. However, in small, thin, or comminuted fragments, screw fixation may increase the risk of fragment splitting or inadequate rotational control. In such situations, a locking plate system combined with suture reinforcement may provide an alternative strategy by distributing load around the fragment and improving resistance to rotational shear. The plate system evaluated in this study may be particularly relevant for cases in which direct screw fixation is not reliable. Still, surgical decisions should also consider fracture morphology, soft-tissue condition, bone quality, implant availability, and surgeon experience.

This study has several limitations. First, the finite element models were reconstructed from the imaging data of a single subject. Anatomical variation, bone mineral density, and fracture morphology were not fully represented. Therefore, the numerical values reported here should not be generalized directly to all patients. Second, the material properties were simplified as homogeneous, isotropic, and linearly elastic. Real bone and ligament tissues are heterogeneous and anisotropic, and their mechanical behavior may vary among individuals (Wang et al., 2025). Such linear simplifications may slightly overestimate peak bone stress and ignore ligament nonlinear stiffening under large tensile deformation. Third, the ligaments were modeled as spring elements, which is a common simplification in ankle finite element studies but cannot fully reproduce the nonlinear and direction-dependent behavior of ligamentous structures. Fourth, only static loading conditions were simulated. *In vivo* ankle loading is dynamic and includes repeated cyclic loading, muscle forces, and complex joint motion. Fatigue behavior and failure risk of the implants were not assessed. Fifth, the interface conditions between implants, bone, and fracture surfaces were simplified. These assumptions may influence the calculated stress and displacement values. Variations in friction coefficients and contact types will directly alter load transfer efficiency and change the absolute magnitude of simulated fragment stress and displacement. Finally, the model was not validated against cadaveric biomechanical testing (Anderson et al., 2008). Further experimental validation is needed to confirm the mechanical trends observed in this study.

## 5 Conclusion

In this finite element study, the novel distal tibiofibular locking plate system demonstrated better biomechanical performance than cannulated screw and suture anchor fixation for Tillaux-Chaput fractures. The composite fixation structure, consisting of plate support, locking screws and suture reinforcement, effectively reduced fragment stress, limited fragment displacement, and improved resistance to rotational shear forces. Notably, Plate-2 yielded the minimum fragment stress across all loading scenarios, yet its implant stress and fragment displacement were not the lowest under every single mechanical condition. These findings suggest that the proposed locking plate system may be a promising fixation option for small or comminuted Tillaux-Chaput fragments with superior comprehensive mechanical performance under most physiological loads.

## Data Availability

The raw data supporting the conclusions of this article will be made available by the authors, without undue reservation.
